# Comparison of the evolution of mechanical ventilation associated pneumonia (VAP) before the implantation of selective digestive decontamination (DDS) and after implantation of pneumonia zero program (NZ)

**DOI:** 10.1186/2197-425X-3-S1-A706

**Published:** 2015-10-01

**Authors:** R Fernández Fernández, L Serrano Fernández, L Camacho Peinado, M Herreros Gonzalo, N Cruza Leganés, M Á Taberna Izquierdo, F Alba García, F Árbol Linde

**Affiliations:** Hospital Nuestra Señora del Prado, Unidad Cuidados Intensivos, Talavera de la Reina, Spain

## Introduction

VAP is the primary infection acquired in Intensive Care Units (ICU). Our goal has been to decrease the state average rate of VAP within 9 episodes per 1,000 days of mechanical ventilation. for this, Spanish Society of Intensive Care Medicine (SEMICYUC), has agreed measures called Pneumonia Zero (NZ) program

## Objectives

We analyze the results before DDS, after DDS´ implantation and after implantation NZ, and compare them with the rest of the country.

## Methods

- Data from the ENVIN registration: 01.01.05 / 01.01.15. ICU with 10 beds.

- We start DDS (in 2009) before the NZ program was recommended by SEMICYUC.

- Import of DDS from a pharmacy which is performed by master formulates.

- Conduct training and educational campaign (presencial and by means of internet) to professionals of ICU (total of 58 people).

- Mandatory measures for the prevention of VAP: proper training in airway management, strict hand hygiene, control of pneumopressure ball(> 20 cmH_2_O), oral hygiene every 6-8 hours, avoid, whenever possible, supine position at 0 °, reduce intubation and / or its duration, prevent the programated change of tubing, humidifiers and tracheal tubes

- Statistical Support ENVIN: Rate/100 patients admitted, 100 days of stay (pDS), 100 patients mechanical ventilation (pMV). N = 3388 pattients admitted.

## Results

- **BEFORE DDS:** 01/01/05-31/01/09. N = 542. VAP 30. 5´54/100 pattients, 8´37/100 DS, 15´08/100 MV. Spain : 5´29/100 pattients, 7´35/100 DS.

- **DDS:** 01/01/09-31/01/11. N = 1163. VAP 6. 0´52/100 pattients, 1´06/100 DS, 2´25/100 MV. Spain : 1´93/100 pattients, 3´14/DS, 8´87/100 MV.

- **NZ :** 01/01/11-31/01/15. N = 1683, VAP 5. 0´3/100 pattients, 0´66/100 DS, 1´47/100 MV. Spain : 1´43/100 pattients, 2´77/100 DS, 5´8/100 MV.

## Conclusions

Our numbers, compared to the rest of Spain, show a lower incidence of VAP. After the application of DDS and the establishment of NZ program, has drastically reduced the incidence of VAP. There is hardly any difference between the rate of VAP obtained after starting DDS and obtained by implanting NZ. We can say that both stuck (DDS and NZ) are adequate to prevent VAP
