# Erratum to: Comparison of the evolution of mechanical ventilation associated pneumonia (VAP) before the implantation of selective digestive decontamination (DDS) and after implantation of pneumonia zero program (NZ)

**DOI:** 10.1186/s40635-016-0082-5

**Published:** 2016-06-13

**Authors:** R. Fernández Fernández, M. Herreros Gonzalo, L. Serrano Fernández, L. Camacho Peinado, N. Cruza Leganés, M. Á. Taberna Izquierdo, F. Alba García, F. Árbol Linde

**Affiliations:** Hospital Nuestra Señora del Prado, Unidad Cuidados Intensivos, Talavera de la Reina, Spain

Unfortunately, after publication of this article [1], it was noticed that the author list order was incorrect. The author María Herreros Gonzalo should have been second in the author list. The corrected author order can be seen above.

## References

[1] Fernández Fernández (2015). Comparison of the evolution of mechanical ventilation associated pneumonia (VAP) before the implantation of selective digestive decontamination (DDS) and after implantation of pneumonia zero program (NZ). Intensive Care Medicine Experimental.

